# Intelligence and cortical morphometry: caveats in brain-behavior associations

**DOI:** 10.1007/s00429-024-02792-6

**Published:** 2024-05-25

**Authors:** John D. Lewis, Vandad Imani, Jussi Tohka

**Affiliations:** 1 Program in Neuroscience and Mental Health, The Hospital for Sick Children Research Institute, 555 University Avenue, Toronto, ON M5G1X8 Canada; 2 A.I. Virtanen Institute for Molecular Sciences, University of Eastern Finland, Neulaniementie 2, 70210 Kuopio, Finland

**Keywords:** Intelligence, Brain size, Morphometry, Confounds, Collinearity

## Abstract

It is well-established that brain size is associated with intelligence. But the relationship between cortical morphometric measures and intelligence is unclear. Studies have produced conflicting results or no significant relations between intelligence and cortical morphometric measures such as cortical thickness and peri-cortical contrast. This discrepancy may be due to multicollinearity amongst the independent variables in a multivariate regression analysis, or a failure to fully account for the relationship between brain size and intelligence in some other way. Our study shows that neither cortical thickness nor peri-cortical contrast reliably improves IQ prediction accuracy beyond what is achieved with brain volume alone. We show this in multiple datasets, with child data, developmental data, and with adult data; we show this with data acquired either at multiple sites, or at a single site; we show this with data acquired with different MRI scanner manufacturers, or with all data acquired on a single scanner; and we show this with fluid intelligence, full-scale IQ, performance IQ, and verbal IQ. But our point is not really even about IQ; rather we proffer a methodological caveat and potential explanation of the discrepancies in previous results, and which applies broadly.

## Introduction

Despite some inconsistencies, a considerable body of research, including sizable meta-analyses, has now shown that brain size correlates with intelligence (Van Valen 1974; Willerman et al. 1991; Wickett et al. 1994; Rushton and Ankney 1996; Vernon et al. 2000; Wickett et al. 2000; Anderson 2003; McDaniel 2005; Witelson et al. 2006; Choi et al. 2008; Luders et al. 2009; Rushton and Ankney 2009; Pietschnig et al. 2015; Gignac and Bates 2017; Cox et al. 2019; Nave et al. 2019). But beyond brain size, things are less clear. There are disturbing discrepancies in the literature on the relation between cortical morphometric measures and intelligence. Some papers have claimed moderate correlations between intelligence and cortical thickness, or peri-cortical contrast (Shaw et al. 2006; Narr et al. 2007; Karama et al. 2009, 2011; Yang et al. 2013; Zhao et al. 2022). Other studies have found conflicting results, or no significant relation between intelligence and any cortical morphometric measure (Menary et al. 2013; Schnack et al. 2015; Brueggeman et al. 2019; Guerdan et al. 2019; Mihalik et al. 2019; Oxtoby et al. 2019; Pohl et al. 2019; Pölsterl et al. 2019; Rebsamen et al. 2019; Valverde et al. 2019; Vang et al. 2019; Wlaszczyk et al. 2019; Zhang-James et al. 2019; Li et al. 2022). Indeed, the failure of all predictive models in *The ABCD Neurocognitive Prediction Challenge* in 2019 was the spark for the current paper. We were surprisd by the poor performance of all 24 teams, given the strength of the relationships previously reported in the literature.

It is unclear what might explain these discrepancies; a number of possibilities have been suggested: (a) differences between scanners, for instance in field strength or scanner manufacturer; (b) differences in the acquisition protocols resulting in differences in scan quality; (c) noise in the MRI data as a result of motion or suboptimal acquisition protocols (Kharabian Masouleh et al. 2019; d) inconsistencies in the MRI data as a result of site differences in multi-site agglomerative data (Kharabian Masouleh et al. 2019; e) differences in the tools used to extract the cortical morphometric measures; (f) differences between samples, e.g. differences in the ages of the subjects (Menary et al. 2013; Schnack et al. 2015), different proportions of males and females (Haier et al. 2005); or (g) noise in the psychometric data due to inconsistencies in the way the data are collected, particularly a problem with agglomerative data (Kharabian Masouleh et al. 2019). All of these are indubitably problematic.

But we suggest that perhaps even more problematic has been the use of multivariate regression models in which the cortical morphometric measure is the dependent variable, the intelligence measure an independent variable, and its coefficient interpreteted as the relation between the intelligence measure and the cortical morphometric measure. It should be noted that the reason that the cortical morphometric measure has been taken as the dependent variable, is that it is not a single measure, but rather a mesh with a different measure at each point, and there are not methods to perform a regression analysis with such a structure as an independent variable; rather, this requires performing the regression analysis separately for each point on the mesh. Such an analysis is, of course possible, but would be quite computationally expensive, and leaves the problem of how to handle the issue of the non-independence of the measures in neighbouring vertices. But, if the cortical morphometric measure, i.e. the mesh of measures, is taken as the dependent variable, the analysis can be run but once, and with the neighbourhood non-independence problem dealt with. But, with this methodology, collinearity is an issue. The model must include brain volume; but brain volume is correlated with intelligence, and when two or more of the independent variables are correlated, the relationships between each of those independent variables and the dependent variable is potentially distorted, making interpretation of their coefficients impossible (Daoud 2017; Alin 2010; Lavery et al. 2019; Leeuwenberg et al. 2021). Avoiding this problem by simply not including variables in the model that should be included, is not a viable solution; that also makes the interpretation of the coefficients of the model impossible.

But collinearity is only a serious issue when two or more independent variables are *highly* correlated. It is unclear to what extent collinearity is an issue at the level of correlation between brain volume and intelligence, typically estimated to be between 0.19 and 0.6 (Wickett et al. 2000; McDaniel 2005; Pietschnig et al. 2015; Nave et al. 2019). We investigate this via models that directly predict the measure of intelligence rather than using it as an independent variable and interpreting its coefficient as the strength of its relation to the morphmetric measure. We assess our ability to predict intelligence on the basis of either of two cortical morphometric measures—cortical thickness and peri-cortical contrast—within several large datasets: (i) the Adolescent Brain Cognitive Development (ABCD) Study consisting of intelligence scores and brain MRI data for more than 8000 children age 9–11 years of age (Casey et al. 2018; ii) the National Institutes of Health Pediatric Dataset, consisting of brain MRI data and measures of intelligence for a representative sample of 216 healthy children and adolescents between the ages of 6 and 20 from the NIH MRI Study of Normal Brain Development (Evans and Group 2006); and (iii) the Enhanced Nathan-Klein Institute - Rockland Sample (Nooner et al. 2012)—commonly known as the NKI-RS data—limited to individuals between 20 and 65 years of age. Thus, we investigate this in child data, data from individuals from middle childhood through adolescence, and in adult data.

For each dataset we predict the measures of IQ using only brain volume, age, sex, and scanner manufacturer; and then again using brain volume, age, sex, scanner manufacturer, and either cortical thickness or peri-cortical contrast. These pairs of results allow us to calculate the improvement in the predictions provided by the inclusion of the cortical morphometric measures. Thus we are not controlling for brain size, or factoring it out, but rather asking what, if anything does the cortical morphometric measure add in terms of prediction accuracy. Previous research has reported results for each of these datasets using the methodology that we are suggesting may be problematic. Thus, if our results agree with these previous results, then we will have shown that there is, in fact, no issue; but if our results are very different, then we will have shown that multi-collinearity is a problem for that methodology.

## Materials and methods

This paper comprises essentially three different studies: we assess IQ prediction in children; IQ prediction in developmental data, i.e. in data from subjects from middle childhood through adolescence; and IQ prediction in adults. The first uses the first visit data from the ABCD study (Casey et al. 2018) ; the second uses the first visit data from the NIH pediatric data (Evans and Group 2006) ; and the third uses data from the Nathan-Klein Institute–Rockland Sample (Nooner et al. 2012)—limited to individuals between 20 and 65 years of age. The datasets are summarized in Table 1 and the subject codes of the participants are included in the supplementary material.^1^

**Table 1:** Overview of the datasets

Dataset	n	$\male /\female$	Age in years (range)	IQ measures
ABCD	3964	2091/1873	10.01 ± 0.61 (9–11)	NIH Toolbox – fluid IQ
NIHPD	383	181/202	11.29 ± 3.64 (6–20)	WASI – FSIQ, PIQ, VIQ
NKI-RS	373	250/123	40.46 ± 14.30 (20–65)	WASI – FSIQ, PIQ, VIQ

WASI: Wechsler Abbreviated Scale of Intelligence ;  IQ: Intelligence Quotient;

FSIQ: Full Scale IQ; PIQ: Performance IQ;  VIQ: Verbal IQ

### The ABCD child data

#### Participants

The ABCD Study is a large multi-site study of brain development and child health in America, funded by the National Institutes of Health (NIH). Researchers at 21 sites across the country track children’s biological and behavioral development. They invited 11,880 children ages 9–11 to participate. The data used here were made available to the participants in the *The ABCD Neurocognitive Prediction Challenge* held in conjunction with the 2019 *Medical Image Computing and Computer Assisted Intervention* conference. The organizers withheld approximately 50% of the data to use as a test dataset. They made available the MRI and standardized fluid intelligence scores for the rest. From what was provided, we used only data that was acquired during the first visit, which left us with 4151 children (ABCD-NP-Challenge 2019).^2^ From these 4151 children, we excluded the data from 187 due to failed MRI pre-processing. Thus we used a cohort of 3964 children, of whom 52.7% were male.

#### Data acquisition

The data were obtained from the NIMH Data Archive (NDA) database 3 as participants in *The ABCD Neurocognitive Prediction Challenge*. Multimodal neuroimaging data, as well as an extensive set of behavioural, cognitive, genetic, and other data 4, were acquired for the ABCD Study. The ABCD Study builds upon imaging protocols from the Pediatric Imaging, Neurocognition Genetics (PING) study (Jernigan et al. 2016) and the Human Connectome Project (HCP) (Van Essen et al. 2013). The study acquired t1-weighted and t2-weighted structural MRI, diffusion MRI, and functional MRI data (Casey et al. 2018). The data were collected on either a Siemens, General Electric (GE), or Philips 3 Tesla scanner, using real-time motion correction and motion monitoring when available; for the t1- and t2-weighted structural MRI, prospective motion correction was used on Siemens and GE scanners. Here, only the t1-weighted structural MRI data were used, together with the measures of intelligence. The t1-weighted acquisition (1 mm isotropic) was a 3D inversion prepared RF-spoiled gradient echo scan with a similar set of parameters across the three different scanner manufacturers (Casey et al. 2018).

The fluid intelligence scores were provided by *The ABCD Neurocognitive Prediction Challenge*; they were determined utilizing the NIH Toolbox Neurocognition battery (Akshoomoff et al. 2013), and demographic factors (e.g., sex and age) were eliminated to remove the effect of confounding variables.

#### Image processing

The ABCD data were retrieved as skull-stripped NIFTI volumes that had been pre-processed with the Minimal Processing pipeline (Hagler et al. 2018) and the NCANDA pipeline (Pfefferbaum et al. 2018) to denoise, correct bias-field inhomogeneities, align to an adult brain atlas, and extract a brain mask. The resulting volumes were then converted to MINC 5 and processed with CIVET (version 2.1; 2016) 6 to extract surfaces at the inner edge of the cortex and at the outer edge of the cortex. The surface at the inner edge of the cortex is produced by fitting a surface to the tissue classified as white-matter; hence it is called the white surface. The surface at the outer edge of the cortex (the pial surface) is produced by moving a copy of the white surface outward until it reaches the external cerebral spinal fluid (CSF); importantly, as it moves outward, vertices can be pulled sideways by elastic forces. Thus, cortical thickness cannot be measured simply as the distance between corresponding vertices in either surface. Instead, the cortical thickness measures were produced by measuring the Laplacian distance between the white surface and the pial surface at each vertex. The Laplacian distance is the length of the path between the pial and white surfaces following the tangent vectors of the streamlines between the two surfaces represented as a Laplacian field (Kim et al. 2005; Jones et al. 2000). This ensures that any misalignment of the vertices in the white and pial surfaces do not introduce error in the thickness measures. To produce the peri-cortical contrast measure, two additional surfaces were generated: a subwhite surface 1 mm inside the white-matter surface; and a suprawhite surface 1 mm outside the white-matter surface. Both of these surfaces were produced by moving the vertices of a copy of the white-matter surface 1 mm along the surface normal—inward for the subwhite surface, and outward for the suprawhite surface. The peri-cortical contrast measures were produced by, at each vertex, dividing the intensity of the t1-weighted image on the subwhite surface by the intensity of the t1-weighted image on the suprawhite surface.

Both the cortical thickness and the peri-cortical contrast measures were then averaged within the regions defined by the AAL atlas, and by each of our down-sampled meshes: our mesh divisions with 160, 640, and 2560 parcels  (described in detail in Lewis et al. (2018)). In predictive settings, it is helpful to reduce the number of predictor variables (although, for example, elastic net penalized linear regression does the selection of variables based on regularization). This is not an issue of computing power, but removing redundant predictors serves the purpose of making predictive models more generalizable. This can be seen as analogous to spatial smoothing in the standard massively univariate modeling, where we can (roughly) approximate that our 2560 mesh division corresponds to smoothing with an 8 mm FWHM kernel. It is a delicate question what is the correct amount of averaging, and that is why we have used several mesh divisions, covering more than the range of spatial smoothing that has been applied in the works that we refer to.

### The NIH developmental data

#### Participants

The NIH Developmental Dataset stems from a multi-site longitudinal project aimed at providing a normative database to characterize healthy brain maturation in relation to behavior (Evans and Group 2006). Data were collected at six pediatric study centers: Boston—Children’s Hospital; Cincinnati—Children’s Hospital Medical Center; Houston—University of Texas Houston Medical School; Los Angeles—Neuropsychiatric Institute and Hospital, UCLA; Philadelphia—Children’s Hospital of Philadelphia (CHOP); and St. Louis—Washington University. The ethics committees of the respective scanning sites approved the study, and informed consent for all subjects was obtained from the subjects, or the children’s parents, for children under 18 years of age. The database includes 433 children from 4 to 22 years of age at enrollment. But only data from the first visit were used here, and only data from healthy subjects without a prior history of medical illnesses with CNS implications, and who were not excluded from the study for other reasons, e.g. having had intra-uterine exposure to substances known or highly suspected to alter brain structure or functions. In this part of our study, we conducted the analysis with the initial visit data from a sample of 383 individuals, ranging from 6 to 20 years of age, comprising 181 males and 202 females.

#### Data acquisition

The NIH Pediatric project acquired t1-weighted, t2-weighted, and PD-weighted images. The data were acquired on either a Siemens or a General Electric 1.5 Tesla scanner. The t1-weighted data were acquired with a 3D RF-spoiled gradient echo acquisition with a repetition time of 22–25 ms, an echo time of 10-11 ms, a flip angle of $30^{\circ }$, a refocusing pulse of $180^{\circ }$, using a sagittal acquisition with a field of view of 256 mm superior-inferior and 204 mm anterior-posterior. The slice thickness was 1.0 mm on Siemens scanners and 1.1$-$1.5 mm on General Electric scanners. The t2- and PD-weighted data were acquired with an axial 2D dual contrast fast spin echo sequence with TR = 3500 ms, TE1 = 15-17 ms, TE2 = 115-119 ms, FOV of 256 mm AP and 224 mm left-right with a 2 mm slice thickness. The images were quality controlled to eliminate scans that contained severe motion artifacts. A total of 324 subjects passed quality control and were used in the processing described below.

Intelligence was measured with the Wechsler Abbreviated Scale of Intelligence (WASI), from which we took three scores: full-scale IQ (FSIQ), performance IQ (PIQ), and verbal IQ (VIQ).

#### Image processing

The MRI images were converted to MINC 7 and processed with CIVET (version 2.1; 2016) 8 to derive measures of cortical thickness and peri-cortical contrast, as described in Section “Image processing”. Each of these measures was then averaged within the regions defined by the AAL atlas, and by each of our down-sampled meshes: our 160, 640, and 2560 mesh divisions.

### The NKI  –RS Adult Data

#### Participants

The Enhanced Nathan-Klein Institute  –Rockland Sample is an ongoing endeavor aimed at collecting a large sample across the lifespan (Nooner et al. 2012). Data collection received ethics approval through both the Nathan Klein Institute and Montclair State University. Written informed consent was obtained from all participants, and in the case of minors, also from their legal guardians. Measures include a wide array of physiological and psychological assessments, genetic information, and advanced neuroimaging. The MRI data are publicly available; the neurocognitive data can be accessed with registration for research purposes. We included data from all subjects who were between 20 and 65 years of age, for whom there were both usable structural imaging data and measures of intelligence. In this part of our study, we used brain MRI data and intelligence scores to conduct an analysis for a cohort of 373 subjects, comprising 250 males with a mean age of 42.29 years and 123 females with a mean age of 36.75 years.

#### Data acquisition

Multimodal neuroimaging data, as well as a large battery of behavioural and cognitive assessments, including the Wechsler Abbreviated Scale of Intelligence (WASI) measures 9, were acquired for the NKI-RS study. We used only the structural MRI and the WASI data. The NKI-RS imaging data were acquired from a single Siemens Magnetom TrioTim 3 Tesla scanner. t1-weighted images were acquired with an MPRAGE sequence (TR = 1900 ms; TE = 2.52 ms; voxel size = 1 mm isotropic). Functional MRI and diffusion imaging data were also collected; but we use only the t1-weighted data.

#### Image processing

The MRI images were converted to MINC 10 and processed with CIVET (version 2.1; 2016) 11 to derive measures of cortical thickness and peri-cortical contrast, as described in Section “Image processing”. Each of these measures was then averaged within the regions defined by the AAL atlas, and by each of our down-sampled parcellations: our 160, 640, and 2560 mesh divisions.

### Predicting IQ

We used each set of brain imaging and demographic variables $\textbf{x}$ to train a predictive model *f* for IQ scores *q*, i.e., so that $q \approx f(\textbf{x})$. Our primary interest was in comparing whether $\textbf{x}$ consisting of cortical thickness or pericortical contrast measures in addition to age, sex, scanner manufacturer—when data were acquired on multiple scanners—and brain volume would allow better predictive models (in the generalization sense) than using just age, sex, scanner manufacturer, and the brain volume of the participant. For the analyses not to be confounded by a particular experimental setup, we used various linear and nonlinear machine learning models (Elastic-net penalized linear regression (ENLR), Random Forest (RF), Support Vector Regression (SVR), and XGBoost Regression (XGR), see Section “Learning algorithms”) with hyperparameters selected via nested cross-validation procedure (see Section “Hyperparameter selection”), and considered various mesh-division schemes for cortical measures (the AAL, and our down-sampled meshes with 160, 640, and 2560 mesh divisions, see Section “Image processing”). The code for the experiments is available at https://github.com/vandadim/IQ_prediction. The overview of the experimental procedure is given in Algorithm 1.

**Algorithm 1:**
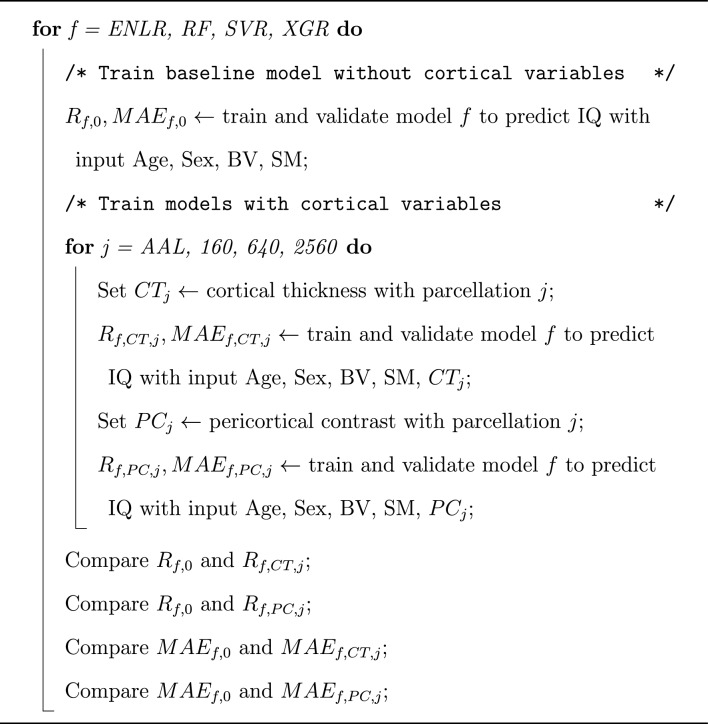
Overview of experiments with each dataset. SM scanner manufacturer; BV brain volume; CT cortical thickness; PC peri-cortical contrast; R correlation coefficient: MAE mean absolute error

#### Learning algorithms

We employed four regression techniques to predict IQ scores: ENLR (Friedman et al. 2010), RF (Breiman 2001), SVR (Chang and Lin 2011), and XGR (Chen and Guestrin 2016). The glmnet library 12 was used for the Elastic-net penalized linear regression model. For the SVR ,^13^ RF ,^14^ and XGBoost 15 models, we used the implementations in the scikit-learn (Pedregosa et al. 2011) library in Python.

#### Validation

In order to quantify the performance of our models, we employed two evaluation metrics: the correlation coefficient (R) between the predicted and observed IQ values and the mean absolute error (MAE) between the observed and predicted IQ scores.

To estimate generalization of MAE and R, we employed a nested tenfold cross-validation (CV) procedure. The nested CV approach involves a double loop, consisting of an inner CV loop for model/hyperparameter selection executed within an outer CV loop to evaluate the trained model. The data were divided into 10 folds (outer loop), and each fold, in turn, was used to evaluate the performance of the model trained on all the other folds. To determine the optimal parameters for each of the 10 outer folds, a 10-fold CV (inner CV loop) was performed. The hyperparameters were determined through the inner CV process, while the performance of the models was evaluated through the outer CV loop to avoid the risk of overfitting due to training on the test data.

We also estimated 95% confidence intervals for the cross-validated correlations using the bootstrap approach that we used in Lewis et al. (2018).

#### Hyperparameter selection

The hyperparameters for the RF, SVR, and XGBoost regression models were determined through a randomized search algorithm by minimizing the MAE using the RandomizedSearchCV function in Python. This was employed instead of a grid search, which exhaustively explores all possible combinations of hyperparameters. The RandomizedSearchCV function was used because it is computationally more efficient and can find good solutions faster than a grid search, especially for high-dimensional search spaces.

For ENLR, the optimal value of the regularization parameter ($\lambda$) was selected through a 10-fold inner cross-validation procedure by minimizing the MAE, using one-dimensional grid-search and a warm start strategy typical with elastic net regression. Additionally, to ensure a balanced contribution from both the ridge and lasso penalties, we set α to the value of 0.5.

The hyperparameters for the Random Forest model included n_estimators (number of trees in the forest), max_depth (maximum depth of each tree), min_samples_split (minimum number of samples required to split an internal node), min_samples_leaf (minimum number of samples required to be at a leaf node), and max_samples (fraction of samples to be drawn from X to train each base estimator). The optimal values of these hyperparameters were found through a randomized search, where the candidate set consisted of n_estimators with values of 100, 1000, and 10,000, max_depth with values ranging from 1 to 10 (in increments of 1), min_samples_split with values of 2, 5, 10, and 15, min_samples_leaf with values of 1, 2, 5, and 10, and max_samples with values ranging from 0.1 to 0.5 (in increments of 0.1).

The hyperparameters for the SVR model included the penalty parameter ‘C’ with values [1, 10, 100, 1000], the kernel type ‘kernel’ with options [‘rbf’, ‘sigmoid’, ‘linear’], the degree of the polynomial kernel function ‘degree’ with values [1, 2, 3, 4, 5, 6], and the kernel coefficient ‘gamma’ with values [0.1, 0.01, 0.001].

The hyperparameters for the Random Forest model included the number of trees ‘n_estimators’ ranging from 100 to 1000 (in increments of 100), the maximum depth of each tree ‘max_depth’ ranging from 1 to 10 (in increments of 1), the learning rate ‘learning_rate’ with values [0.1, 0.01, 0.001], the minimum number of samples required to be in a child node ‘min_child_weight’ ranging from 1 to 6 (in increments of 1), the regularization term ‘gamma’ with values ranging from 0 to 0.9 (in increments of 0.1), the fraction of the training data to be used for building each tree ‘subsample’ ranging from 0.1 to 0.9 (in increments of 0.1), and the fraction of columns to be randomly sampled for each split ‘colsample_bytree’ ranging from 0.1 to 0.9 (in increments of 0.1).

## Results

The IQ prediction results for the ABCD child data are presented in section “The ABCD child data results”; the results for the NIH developmental data are presented in section “The NIH developmental data results”; the results for the NKI-RS adult data are presented in section “The NKI-RS adult data results”.

### The ABCD child data results

The summarized results for the ABCD Study data are given in Tables 2 and 3. The full set of results are presented in the supplementary material. Table 2 presents the means, standard deviations, and ranges of the changes in the correlations between the predicted FIQ and the actual FIQ, across learning models and mesh-divisions, when the cortical morphometric measures are included in the model compared to the model without the cortical morphometric measure; and also the means, standard deviations, and ranges of the mean absolute errors for the same. These results are presented for the model with cortical thickness; and for the model with peri-cortical contrast. Note that for both cortical morphometric measures, the results show, on average, a small improvement in prediction accuracy; but the ranges for both the correlation and the change in mean absolute error indicate that a decrease in accuracy is also possible.

**Table 2:** ABCD. Results for regression models for predicting FIQ based on either only age, sex, scanner manufacturer (SM), and brain volume (BV); or the same, but also with either cortical thickness (CT) or peri-cortical contrast (PC). Here we present the mean and standard deviations of the change in the correlations between the predicted FIQ and the actual FIQ when the cortical morphometric measures are included in the model, across learning models and mesh-divisions; the means and standard deviations for the change in mean absolute error (MAE) for these differences are also presented. The range for each of these measures is presented in square brackets beneath. Note that for both cortical morphometric measures, on average, their inclusion yields a small increase in the accuracy of the predictions relative to the model with only brain volume, i.e. they show a positive value of $\mu$($\Delta$ R), and a negative value for $\mu$($\Delta$ MAE). But note also that the standard deviations and ranges for both the change in correlation and the mean absolute error indicate that prediction accuracy may also decrease

FIQ	$\mu (\Delta \hbox { R})\,\pm \,\sigma$	$\mu (\Delta \hbox { MAE})\,\pm \,\sigma$
	[min($\Delta$ R) , max($\Delta$ R)]	[min($\Delta$ MAE) , max($\Delta$ MAE)]
Age, Sex, BV, SM, CT	0.089 ± 0.051	$-$0.020 ± 0.020
	[$-$0.014 , 0.149]	[$-$0.055 , 0.005]
Age, Sex, BV, SM, PC	0.075 ± 0.042	$-$0.014 ± 0.015
	[$-$0.016 , 0.125]	[$-$0.042 , 0.007]

Table 3 presents the results for the method and mesh-division that yielded the median change in the correlation between the predicted FIQ and the actual FIQ relative to the same model but without the inclusion of the cortical morphometric measure.

**Table 3:** ABCD. Results for regression models for predicting FIQ based on either only age, sex, scanner manufacturer (SM), and brain volume (BV); or the same, but also with either cortical thickness (CT) or peri-cortical contrast (PC). Here we present the results of the method and mesh-division that yielded the median change in the correlation between the predicted FIQ and the actual FIQ when the cortical morphometric measures are included in the model relative to the same model but without the inclusion of the cortical morphometric measure. We report the improvement in the correlation and the confidence intervals for that change, and the change in the mean absolute error (MAE) and the confidence intervals for that change. The confidence intervals are presented in parentheses beneath those measures of improvement. Note that for both cortical morphometric measures, their inclusion yields a small increase in prediction accuracy; but note also that the confidence intervals for the change in mean absolute error vary from negative to positive for both measures

FIQ	Method	Parc	$\Delta$ R	$\Delta$ MAE
			( lo CI , hi CI )	( lo CI , hi CI )
Age,Sex,BV,SM,CT	glmnet	160	0.107	$-$0.025
			( 0.065 , 0.146 )	( $-$0.056 , 0.008 )
Age, Sex, BV, SM, PC	Xgboost	AAL	0.091	$-$0.027
			( 0.048 , 0.133 )	( $-$0.061 , 0.009 )

The results for each cortical morphometric measure are presented as the change in the correlation between the predicted FIQ and the actual FIQ relative to the version of the model without the inclusion of the cortical morphometric measure, and likewise the change in the MAE yielded by inclusion of the cortical morphometric measure. Table 3 also provides the confidence intervals for both the change in the correlation and the change in mean absolute error yielded by the inclusion of the cortical morphometric measures. Note that the confidence intervals for the mean absolute error for both cortical morphometric measures vary from negative to positive indicating that a decrease in accuracy is also possible.

### The NIH developmental data results

The summarized results for the NIH developmental data are given in Tables 4 and 5. The full set of results are presented in the supplementary material. Table 4 presents, for each of FSIQ, PIQ, and VIQ, the means and standard deviations of the correlations between the predicted IQ and the actual IQ, across learning models and mesh-divisions; and also the mean and standard deviations of the mean absolute errors for the same.

**Table 4:** NIH developmental data. Results for the regression models for predicting FSIQ, PIQ, and VIQ based on either only age, sex, scanner manufacturer (SM), and brain volume (BV); or the same, but also with either cortical thickness (CT) or peri-cortical contrast (PC). Here we present the mean and standard deviations of the change in the correlations between the predicted IQ and the actual IQ when the cortical morphometric measures are included in the model, across learning models and mesh-divisions; the means and standard deviations for the change in mean absolute error (MAE) for these differences are also presented. The range for each of these measures is presented in square brackets beneath. Note that for both cortical morphometric measures, and for all three IQ measures, on average, their inclusion yields a small decrease in the accuracy of the predictions relative to the model with only brain volume. But note also that, with the exception of FSIQ with cortical thickness, the standard deviations and the range of both the change in correlation and the change in mean absolute error suggest that an improvement in prediction accuracy is possible, but will be unreliable. For FSIQ with cortical thickness, both the standard deviations and the ranges of the change in correlation and in mean absolute error indicate that only a decrease in prediction accuracy is possible

FSIQ	$\mu$($\Delta$ R) ± $\sigma$	$\mu$($\Delta$ MAE) ± $\sigma$
	[min($\Delta$ R) , max($\Delta$ R)]	[min($\Delta$ MAE) , max($\Delta$ MAE)]
Age, Sex, BV, SM, CT	$-$0.117 ± 0.067	0.232 ± 0.105
	[$-$0.239 , $-$0.020]	[0.054 , 0.422]
Age, Sex, BV, SM, PC	$-$0.007 ± 0.055	0.026 ± 0.168
	[$-$0.129 , 0.083]	[$-$0.409 , 0.306]
PIQ		
Age, Sex, BV, SM, CT	$-$0.118 ± 0.068	0.112 ± 0.092
	[$-$0.204 , 0.003]	[$-$0.041 , 0.238]
Age, Sex, BV, SM, PC	$-$0.025 ± 0.047	0.057 ± 0.091
	[$-$0.119 , 0.080]	[$-$0.155 , 0.231]
VIQ		
Age, Sex, BV, SM, CT	$-$0.097 ± 0.040	0.090 ± 0.127
	[$-$0.168 , $-$0.025]	[$-$0.047 , 0.492]
Age, Sex, BV, SM, PC	$-$0.031 ± 0.068	$-$0.010 ± 0.113
	[$-$0.222 , 0.072]	[$-$0.268 , 0.178]

These results are presented for the model with cortical thickness and for the model with peri-cortical contrast. Note that for FSIQ and PIQ, for both cortical morphometric measures, their inclusion, on average, yields a reduction in the accuracy of the predictions relative to the model with only brain volume; for VIQ, the inclusion of cortical thickness, on average, yields a reduction in the accuracy of the predictions relative to the model with only brain volume, while the inclusion of peri-cortical contrast, on average, yields a slight reduction in the correlation between predicted VIQ and actual VIQ, but a slight decrease in mean absolute error. But also note that, for peri-cortical contrast, for all three IQ measures, the ranges of both the change in correlation and the change in mean absolute error vary from negative to positive, indicating a possibility of an increase in prediction accuracy; and note that that is not the case for cortical thickness.

Table 5 presents, for each of FSIQ, PIQ, and VIQ, the results for the method and mesh-division that yielded the median improvement in the correlation between the predicted IQ and the actual IQ relative to the same model but without the inclusion of the cortical morphometric measure. The results for both cortical morphometric measures are presented as the change in the correlation between the predicted IQ and the actual IQ relative to the version of the model without the inclusion of the cortical morphometric measure, and likewise the change in the mean absolute error yielded by inclusion of the cortical morphometric measure. Note that for FSIQ and PIQ, the inclusion of either cortical morphometric measure yields a reduction in the accuracy of the predictions relative to the model without it; but note that, in both cases, the confidence intervals indicate that an increase in prediction accuracy is possible. The same is true for VIQ with cortical thickness; but for peri-cortical contrast, the change in the mean absolute error is negative, though the confidence interval admits the possibility of an increase.

**Table 5:** NIH developmental data. Results for regression models for predicting FSIQ, PIQ, and VIQ based on either only age, sex, scanner manufacturer (SM), and brain volume (BV); or the same, but also with either cortical thickness (CT) or peri-cortical contrast (PC). Here we present the results of the method and mesh-division that yielded the median improvement in the correlation between the predicted IQ and the measured IQ when the cortical morphometric measures are included in the model relative to the same model but without the inclusion of the cortical morphometric measure. We report the improvement in the correlation and the confidence intervals for that improvement, and the improvement in the mean absolute error (MAE) and the confidence intervals for that improvement. The confidence intervals are presented in parentheses beneath those measures of improvement. Note that for both cortical morphometric measures, their inclusion yields a small decrease in prediction accuracy; but note also that the confidence intervals for both the change in correlation and the change in mean absolute error vary from negative to positive for both measures; so a small increase in prediction accuracy is possible

FSIQ	Method	Parc.	$\Delta$ R	$\Delta$ MAE
			( lo CI , hi CI )	( lo CI , hi CI )
4 Age, Sex, BV, SM, CT	randomF	640	$-$0.106	0.185
			( $-$0.240 , 0.049 )	( $-$0.995 , 1.135 )
Age, Sex, BV, SM, PC	glmnet	640	$-$0.003	0.086
			( $-$0.123 , 0.149 )	( $-$1.058 , 1.012 )
PIQ				
Age, Sex, BV, SM, CT	SVR	640	$-$0.137	0.064
			( $-$0.274 , 0.031 )	( $-$1.062 , 1.173 )
Age, Sex, BV, SM, PC	Xgboost	640	$-$0.027	0.231
			( $-$0.165 , 0.114 )	( $-$0.943 , 1.250 )
VIQ				
Age, Sex, BV, SM, CT	Xgboost	2560	$-$0.105	0.089
			( $-$0.240 , 0.044 )	( $-$1.181 , 1.201 )
Age, Sex, BV, SM, PC	randomF	640	$-$0.029	$-$0.046
			( $-$0.153 , 0.111 )	( $-$1.339 , 1.106 )

### The NKI-RS adult data results

The summarized results for the NKI-RS adult data are given in Tables 6 and 7. The full set of results are presented in the supplementary material. Table 6 presents, for each of FSIQ, PIQ, and VIQ, the means, standard deviations, and ranges of the correlations between the predicted IQ and the actual IQ, across learning models and mesh-divisions.

**Table 6:** NKI –RS. Results for the regression models for predicting FSIQ, PIQ, and VIQ based on either only age, sex, and brain volume (BV); or the same, but also with either cortical thickness (CT) or peri-cortical contrast (PC). Here we present the mean and standard deviations of the change in the correlations between the predicted IQ and the actual IQ when the cortical morphometric measures are included in the model, across learning models and mesh-divisions; the means and standard deviations for the change in mean absolute error (MAE) for these differences are also presented. The range for each of these changes is presented in square brackets beneath. Note that for both cortical morphometric measures, for all three IQ measures, on average, their inclusion yields a small increase in the accuracy of the predictions relative to the model with only brain volume. But note also that the standard deviations and the range of both the change in correlation and the change in mean absolute error suggest that this improvement in prediction accuracy is unreliable

FSIQ	$\mu$($\Delta$ R) ± $\sigma$	$\mu$($\Delta$ MAE) ± $\sigma$
	[min($\Delta$ R) , max($\Delta$ R)]	[min($\Delta$ MAE) , max($\Delta$ MAE)]
Age, Sex, BV, CT	0.052 ± 0.077	$-$0.100 ± 0.200
	[$-$0.084 , 0.193]	[$-$0.413 , 0.361]
Age, Sex, BV, PC	0.017 ± 0.051	$-$0.037 ± 0.089
	[$-$0.076 , 0.111]	[$-$0.205 , 0.115]
PIQ		
Age, Sex, BV, CT	0.031 ± 0.090	$-$0.006 ± 0.179
	[$-$0.074 , 0.205]	[$-$0.296 , 0.259]
Age, Sex, BV, PC	0.028 ± 0.078	$-$0.061 ± 0.142
	[$-$0.091 , 0.154]	[$-$0.256 , 0.198]
VIQ		
Age, Sex, BV, CT	0.064 ± 0.096	$-$0.130 ± 0.211
	[$-$0.119 , 0.255]	[$-$0.491 , 0.284]
Age, Sex, BV, PC	0.027 ± 0.095	$-$0.083 ± 0.164
	[$-$0.111 , 0.235]	[$-$0.293 , 0.225]

These results are presented for the model with cortical thickness; and for the model with peri-cortical contrast. Note that for all three measures, with both cortical morphometric measures, on average, there is a small increase in prediction accuracy; but note also that in all cases, both the standard deviations and the ranges admit the possibility of a decrease in prediction accuracy.

Table 7 presents, for each of FSIQ, PIQ, and VIQ, the results for the method and mesh-division that yielded the median improvement in the correlation between the predicted IQ and the actual IQ relative to the same model but without the inclusion of the cortical morphometric measure.

**Table 7:** NKI –RS. Results for regression models for predicting FSIQ, PIQ, and VIQ based on either only age, sex, and brain volume (BV); or the same, but also with either cortical thickness (CT) or peri-cortical contrast (PC). Here we present the results of the method and mesh-division that yields the median change in the correlation between the predicted IQ and the actual IQ when the cortical morphometric measures are included in the model. We report the change in the correlation and the confidence intervals for that change, and the change in the mean absolute error (MAE) and the confidence intervals for that change. The confidence intervals are presented in parentheses beneath those measures of change. Note that for both cortical morphometric measures, their inclusion yields a mixed result: a small increase in prediction accuracy for FSIQ with either cortical morphometric measure; a small increase in prediction accuracy for PIQ and VIQ with peri-cortical contrast; a small decrease in prediction accuracy for PIQ with cortical thickness; and an increased correlation between predicted VIQ and actual VIQ with cortical thickness, but also an increase in the mean absolute error.; but note also that, except for VIQ with peri-cortical contrast, the confidence intervals for both the change in correlation and the change in mean absolute error vary from negative to positive for both measures

FSIQ	Method	Parc.	$\Delta$ R	$\Delta$ MAE
			( lo CI , hi CI )	( lo CI , hi CI )
Age, Sex, BV, SM, CT	Xgboost	AAL	0.050	$-$0.025
			( $-$0.072 , 0.167 )	( $-$0.416 , 0.399 )
Age, Sex, BV, SM, PC	glmnet	640	0.016	$-$0.050
			( $-$0.122 , 0.142 )	( $-$0.358 , 0.279 )
PIQ				
Age, Sex, BV, SM, CT	Xgboost	AAL	$-$0.012	0.206
			( $-$0.162 , 0.123 )	( $-$0.244 , 0.681 )
Age, Sex, BV, SM, PC	randomF	2560	0.037	$-$0.114
			( $-$0.092 , 0.174 )	( $-$0.366 , 0.140 )
VIQ				
Age, Sex, BV, SM, CT	glmnet	AAL	0.077	0.045
			( $-$0.051 , 0.209 )	( $-$0.190 , 0.277 )
Age, Sex, BV, SM, PC	randomF	2560	0.034	$-$0.234
			( $-$0.087 , 0.161 )	( $-$0.484 , $-$0.003 )

The results for both cortical morphometric measures are presented as the change in the correlation between the predicted IQ and the actual IQ relative to the version of the model without the inclusion of the cortical morphometric measure, and likewise the change in the mean absolute error yielded by inclusion of the cortical morphometric measure. Note that for both cortical morphometric measures, the results show a small improvement in prediction accuracy, but the confidence intervals indicate that a decrease in prediction accuracy is also a possibility.

## Discussion

In contrast to some previously published results, we have shown that cortical morphometric measures contribute little to IQ prediction. We showed this in multiple datasets, with child data, developmental data, and with adult data; we showed this with data acquired either at multiple sites, or at a single site; we showed this with data acquired with different MRI scanner manufacturers, or with all data acquired on a single scanner; we showed this with fluid intelligence, full-scale IQ, performance IQ, and verbal IQ; and we showed this with cortical thickness and with peri-cortical contrast. Our results suggest that the apparent contradiction with earlier publications claiming to have shown a modest relation between IQ and cortical morphometric measures, such as cortical thickness, was likely due either to a failure to fully remove the contribution of brain volume, and other confounding factors, or to collinearity between the intelligence measure and one or more of the other predictors within a multivariate regression model with the cortical morphometric measure as the dependent variable. We predicted IQ from brain volume, age, sex, and scanner manufacturer (in cases in which multiple scanners were involved), and compared those results to the predictions made from the same variables plus one of the cortical morphometric measures. We showed that the inclusion of either of the cortical morphometric measures made very little difference to the predictions, and any difference was unreliable, with different models producing either small reductions or increases in predictive accuracy.

For the ABCD data, consisting of data from 9 to 11 year-old children, on average, over all models and parcelletion schemes, cortical thickness or peri-cortical contrast boosted the correlation between predicted fluid intelligence and actual fluid intelligence by very little, and similarly reduced the mean absolute error only slightly. For both cortical thickness and peri-cortical contrast, the range of outcomes for both the change in the correlation between predicted fluid IQ and actual fluid IQ, and for the change in the mean absolute error varied from negative to positive indicating that inclusion of the cortical morphometric measure might either increase or decrease prediction accuracy.

For the NIH developmental data, consisting of data from children and adolescents ranging from 6 years of age to 20 years of age, for both cortical morphometric measures, and for FSIQ, PIQ, and VIQ, the inclusion of either cortical morphometric measure yielded, on average, across all models and mesh-division schemes, a decrease in the correlation between predicted IQ and actual IQ; and in all cases except VIQ with peri-cortical contrast, on average, the mean absolute error increased with the inclusion of either cortical morphometric measure. And it is noteworthy that, with the exception of FSIQ with cortical thickness, as with the ABCD data, for both cortical thickness and peri-cortical contrast, the range of outcomes for both the change in the correlation between predicted IQ and actual IQ, and for the change in the mean absolute error, varied from negative to positive indicating that the cortical morphometric measure might either increase or decrease prediction accuracy. For FSIQ with cortical thickness, both the standard deviations and the range of the change in correlation between predicted and actual IQ indicate that only a decrease in prediction accuracy is possible.

For the NKI –RS data, limited to individuals between 20 and 65 years of age, for both cortical morphometric measures, and for FSIQ, PIQ, and VIQ, the inclusion of either cortical morphometric measure yielded, on average, across all models and mesh-division schemes, a small increase in the correlation between predicted IQ and actual IQ, and a small decrease in the mean absolute error; but in all cases, both the standard deviation of those measures and the range indicated that both increased and decreased prediction accuracy were possible.

In sum, we have shown, with child data, developmental data, and with adult data, that either cortical morphometric brain measure contributes little to IQ prediction over what can be predicted from age, sex, scanner manufacturer, and brain volume. On average, over various learning models and mesh-divisions, for both cortical thickness and peri-cortical contrast, the change in prediction accuracy for all IQ measures, in all datasets, might be either a small increase in prediction accuracy, or a decrease; neither outcome was found to be reliable. Additionally, we found that no learning model, and no mesh-division, reliably produced the best outcome.

These results are in line with those of *The ABCD Neurocognitive Prediction Challenge* held in conjunction with the 2019 *Medical Image Computing and Computer Assisted Intervention* conference. That challenge invited researchers to predict fluid intelligence from the ABCD t1-weighted MRI. The fluid intelligence data of approximately 50% of the subjects were provided. Confounding factors were removed from the data, so it was the residual fluid intelligence that was provided. The challenge was to predict the residual fluid intelligence for the remainder of the subjects. The accuracy of each entry was assessed on the predictions made for the subjects whose residual fluid intelligence was not provided. Twenty four teams competed in that challenge. The results suggested that residual fluid intelligence cannot be predicted by features derived from MRI images even with state-of-the-art prediction algorithms, including deep learning. Notably, none of the entries produced predictions for the test subjects that were at all impressive in terms of correlation and mean square error. The winning entry (Mihalik et al. 2019) had a correlation between predicted residual fluid intelligence and the actual residual fluid intelligence, for the test subjects, of only 0.03.

In contrast to our ABCD results, and to the results from the *ABCD Neurocognitive Prediction Challenge*, Zhao et al. (2022) report a relation between cortical thickness and fluid IQ with high *t*-values. There are several important differences between the methods used by Zhao et al. (2022) and the methods used here and by the participants of the *ABCD Neurocognitive Prediction Challenge* that underlie these differences in the results. Most importantly, Zhao et al. (2022) are not predicting IQ; they are predicting cortical thickness, with IQ as one of the predictors, and then inferring the relation between cortical thickness and IQ from the coefficient associated with the IQ predictor. These are two very different things. This latter approach is typical in massively univariate neuroimaging analyses, but can be problematic. As mentioned above, the omission or inclusion of a covariate which is collinear with IQ will distort the coefficient associated with IQ, and thus the inferred relation between IQ and the cortical metric. In the case at hand, the omission of brain volume in Zhao et al.’s model, which is known to be strongly related to IQ, creates the illusion of a strong relation between cortical thickness and IQ. Additionally, Zhao et al.’s model includes only linear relationships with the covariates, which is known to be inadequate. Thus there are multiple reasons to doubt the result reported by Zhao et al. (2022). And it is interesting to note that Zhao et al. (2022) did not mention the conflicting results from the *ABCD Neurocognitive Prediction Challenge*, i.e. Brueggeman et al. (2019); Guerdan et al. (2019); Mihalik et al. (2019); Oxtoby et al. (2019); Pohl et al. (2019); Pölsterl et al. (2019); Rebsamen et al. (2019); Valverde et al. (2019); Vang et al. (2019); Wlaszczyk et al. (2019); Zhang-James et al. (2019).

Both Karama et al. (2009) and Shaw et al. (2006), like Zhao et al. (2022), rather than predicting IQ, predicted cortical thickness with IQ as one of the covariates. And also like Zhao et al. (2022), both Karama et al. (2009) and Shaw et al. (2006) omitted brain volume from their analyses. Thus, once again, the omission of brain volume, despite its known relation to IQ, created the illusion of a strong relation between cortical thickness and IQ. Shaw et al. (2006) acknowledged the existence of “a modest correlation (r = 0.3) between psychometric measures of intelligence and total brain volume", but nonetheless went on to omit brain volume from their analysis; but they did explore non-linear age effects, and reported a non-linear relationship with IQ. Karama et al. (2009), on the other hand, considered only linear relationships with the covariates, including age.

Both Narr et al. (2007) and Karama et al. (2011), also rather than predicting IQ, predict cortical thickness with IQ as one of the covariates ; but both include brain volume as a covariate in their analyses; but like Zhao et al. (2022) and Karama et al. (2009), they consider only linear relationships with the covariates. So, in both of these cases, the issue of collinearity more clearly rears its head ; the correlation between the intelligence measure and brain volume causes their coefficients to be distorted, making interpretation of their coefficients impossible (Daoud 2017; Alin 2010; Lavery et al. 2019; Leeuwenberg et al. 2021). And Karama et al. (2011) utilized the general factor of intelligence, *g*, which is even more strongly correlated with brain volume than are the WASI measures of IQ (Wickett et al. 2000), and thus potentially causes their coefficients to be more distorted, and so more impossible to interpret (Daoud 2017; Alin 2010; Lavery et al. 2019; Leeuwenberg et al. 2021).

We conclude that previous reports of modest relationships between cortical morphometric measures and measures of intelligence have been mistaken. Mostly they have been produced using multivariate regression models with the cortical morphometric measure as the dependent variable, and the measure of intelligence as an independent variable, and then the coefficient of the measure of intelligence interpreted as the strength of the relationship between it and the cortical morphometric measure; such methods rely on the assumption that all predictor variables in the model are uncorrelated, if the coefficients of the individual predictors are to be interpreted. Since brain size and intelligence are known to be correlated, these methods prohibit the interpretation of the coefficient of the intelligence predictor. The omission of predictors that are correlated with the intelligence measures is not a solution to the problem ; that simply produces an invalid model. Further, often only the linear relationships between the covariates and the dependant variable are considered, despite that some of these covariates, e.g. age, are known to have a nonlinear relationship with the dependant variable. Thus, we conclude that previous reports of modest relationships between cortical morphometric measures and measures of intelligence that have been derived via such methods are illusory.

With multiple methods that predict IQ, rather than the reverse, we have shown, with child data, developmental data, and with adult data, that both cortical thickness and peri-cortical contrast contribute little to IQ prediction over what can be predicted from age, sex, scanner manufacturer, and brain volume. And moreover, we have shown that even a claim of a small contribution must be accompanied by the caveat that it will be unreliable.

But the results here, and this conclusion, must also be caveated. Although the datasets in this study are large in neuroimaging, the number of participants can still be considered small for the tree-based machine learning methods applied (Random Forest and XGBoost), and we acknowledge that with larger number of participants more accurate IQ predictions could be obtained. In addition, it is well recognized that the cross-validation or (or any counting-method based) error estimate comes with a large variance (Dougherty 2001), especially when there are a large number of predictor variables (Tohka et al. 2016) and hyper-parameters must be selected within nested cross-validation (Varoquaux 2018). However, we have provided estimates of confidence intervals for predictive accuracy. Also, we have utilized standard learning algorithms that are not crafted for the purpose of MRI-based IQ-prediction and we cannot exclude the possibility that algorithms crafted for this specific purpose would produce better IQ predictions. However, the results of *The ABCD Neurocognitive Prediction Challenge* seem to suggest that specialized learning algorithms would not do any better. Lastly, in the following appendix (Appendix: Simulated data experiment) we demonstrate that, even with the sample sizes that we have used here, if there were a reasonably strong relation, independent of confounds, of cortical thickness or pericortical contrast to an intelligence measure, in any region of the cortex, we would have been able to detect it.

Finally, we note that we believe that what we have shown here should be understood not as a criticism of previous results relating cortical metrics to IQ, but much more broadly; the methodological error that we have highlighted here is in no way specific to IQ, or any measure of intelligence. We intend this paper to direct researchers investigating the relation between any cortical morphometric measure and any behavioural measure to avoid this methodological error.

## Data Availability

The ABCD neuroimaging and intelligence data used in the preparation of this article were obtained from the Adolescent Brain Cognitive Development (ABCD) Study (https://abcdstudy.org). The residualized fluid intelligence data were prepared the organizers of *The ABCD Neurocognitive Prediction Challenge*, which was held in conjunction with the 2019 *Medical Image Computing and Computer Assisted Intervention* conference. The data were made available through the NIMH Data Archive (NDA). The NDA study specifying the data used is https://doi.org/10.15154/1528740. The ABCD Study is a multisite, longitudinal study designed to recruit more than 10,000 children age 9–10 and follow them over 10 years into early adulthood. The NIHPD neuroimaging and intelligence data used in the preparation of this article were obtained from the NIH Pediatric MRI Data Repository created by the NIH MRI Study of Normal Brain Development. The data were made available through the NIMH Data Archive (NDA). Dataset identifier(s): NIMH Data Archieve Collection ID 1151 and NDAR study is https://doi.org/10.15154/1528740. The neuroimaging data for the NKI-RS dataset are publicly available, and full phenotypic data, including the IQ scores, can be requested through http://fcon_1000.projects.nitrc.org/indi/enhanced/access.html. Our code is available at https://github.com/vandadim/IQ_prediction. The software packages used are described in Section 2.4.1.

## Appendix: Simulated data experiment

We conducted a sanity check experiment to confirm that we would be able to detect it if there were to be a strong and independent (from confounds) relation of cortical thickness or pericortical contrast to an intelligence measure. For this experiment, we considered only the AAL parcellation. We replaced cortical thickness or pericortical contrast with a new variable constructed based on FSIQ (NIHPD, NKI-RS) or Fluid IQ score (ABCD). We denote the new variable $\textbf{x} = [x_1, \ldots , x_N]$, where

$$\begin{aligned} x_j = z_j + cr_j, \end{aligned}$$

where $z_j$ is the FSIQ or the fluid intelligence score of the participant *j* z -scored to have a mean of 0 and a standard deviation of 1, $r_j$ is a random number drawn from standard normal distribution and *c* is the constant specifying the strength of the relation (we used the values $c =1$ and $c=2$). *N* is the number of participants. Thus, the expected value of the correlation between the new variable and IQ score is approximately 0.71 ($c=1$) or 0.45 ($c=2$), that is in addition to any actual relation between the data and the IQ scores. We then predicted FSIQ or fluid intelligence based on this data. The results of this experiment, summarized in Table 8 and reported in the supplementary tables, confirmed that indeed we would find this strong relation.

**Table 8:** Simulated data. Results for regression models with simulated datasets constructed based on ABCD, NIHPH, and NKI-RS data with $c=1$ or $c=2$. We report here the average of cross-validated correlation and its standard deviation across the four learning algorithms studied. These measures should be compared to expected correlation values of 0.71 ($c=1$) and 0.45 ($c=2$) of the simulated effect. We do not report MAE for this experiment as it is not meaningful and challenging to interpret. The correlations show that the machine learning algorithms are able to find the simulated effects, do so better with the larger sample-size of the ABCD data and there is significant variation across the learning algorithms and specific simulated setups

c	predictors	$\mu$(R) ± $\sigma$		
		ABCD	NIHPD	NKI-RS
c=1	Age, Sex, BV, PC	0.791 $\pm$ 0.028	0.667 $\pm$ 0.053	0.599 $\pm$ 0.082
c=2	Age, Sex, BV, PC	0.422 $\pm$ 0.014	0.381 $\pm$ 0.061	0.381 $\pm$ 0.076
c=1	Age, Sex, BV, CT	0.793 $\pm$ 0.026	0.636 $\pm$ 0.053	0.688 $\pm$ 0.041
c=2	Age, Sex, BV, CT	0.564 $\pm$ 0.020	0.299 $\pm$ 0.114	0.271 $\pm$ 0.087

## Footnotes

1 https://github.com/vandadim/IQ_prediction. 10.5281/zenodo.11121851.

2 https://sibis.sri.com/abcd-np-challenge/.

3 https://nda.nih.gov.

4 For details, see  https://abcdstudy.org/scientists/.

5 https://www.bic.mni.mcgill.ca/ServicesSoftware/MINC.

6 http://www.bic.mni.mcgill.ca/ServicesSoftware/CIVET-2-1-0-Introduction.

7 https://www.bic.mni.mcgill.ca/ServicesSoftware/MINC.

8 http://www.bic.mni.mcgill.ca/ServicesSoftware/CIVET-2-1-0-Introduction.

9 For details, see  https://fcon_1000.projects.nitrc.org/indi/enhanced/.

10 https://www.bic.mni.mcgill.ca/ServicesSoftware/MINC.

11 http://www.bic.mni.mcgill.ca/ServicesSoftware/CIVET-2-1-0-Introduction.

12 The glmnet library is available at https://glmnet.stanford.edu/articles/glmnet.html which was used through a Python wrapper https://pypi.org/project/glmnet/.

13 The SVR function is available in the support vector machine library of scikit-learn, which can be accessed at https://scikit-learn.org/stable/modules/generated/sklearn.svm.SVR.html.

14 The ’RandomForestRegressor’ function is available in the ensemble library of scikit-learn, which can be accessed at https://scikit-learn.org/stable/modules/generated/sklearn.ensemble.RandomForestRegressor.html.

15 The XGBoost library, which includes the XGBRegressor function, can be found at https://xgboost.readthedocs.io/en/stable/python/python_api.html.
